# Central Nervous System Xanthoma Disseminatum: Response to 2CdA in an Adolescent

**DOI:** 10.1155/2022/9906668

**Published:** 2022-07-22

**Authors:** Patrick DeMoss, Nancy Tang, Kristen Yeom, Audris Chiang, Ann L. Marqueling, Michael R. Jeng

**Affiliations:** Departments of Pediatrics (PD, NT, MJ), Radiology (KY), and Dermatology (AC, AM), Stanford University School of Medicine, Stanford, California, USA

## Abstract

Xanthoma disseminatum is a normolipemic non-Langerhans cell histiocytosis characterized by red-brown rubbery papules of the skin which coalesce into plaque-like lesions with symmetric involvement of face, flexor, and intertriginous areas. Less commonly, xanthoma disseminatum may affect mucosal linings, abdominal organs, and the central nervous system, leading to endocrinopathies. We report a 12-year-old adolescent with mucosal, central nervous system, and painful cutaneous lesions, further complicated by diabetes insipidus and amenorrhea. Treatment with 2-chlorodeoxyadenosine led to relief of pain and significant improvement of mucosal, central nervous system, and cutaneous lesions, with subsequent restoration of menstrual cycles.

## 1. Introduction

Xanthoma disseminatum (XD) is a non-Langerhans cell histiocytosis (nLCH), characterized by a yellow-brown, papulonodular rash of the axilla, eyelids, groin, neck, trunk, and face, initially presenting as isolated nodules coalescing into plaques over time [1]. Mucosal (larynx, pharynx, mouth, trachea, conjunctiva and cornea, epiglottis, and tongue) lesions arise in approximately 60% of patients with XD [2]. Hypothalamic-pituitary infiltration, causing endocrinopathies such as diabetes insipidus (DI), occurs in approximately 40% of patients, while osseous, intra-abdominal visceral, and central nervous system (CNS) involvement is less common [1]. Many patients are diagnosed based on physical examination alone.

There is no standard of care established for the treatment of XD. Case reports in the literature describe responses to individualized treatments, i.e., observation [1], cyclophosphamide [1], clofibrate [3], steroid monotherapy [3, 4], steroids with excision and electrocautery [5], vinblastine monotherapy [6], acitretin and thalidomide [7], azathioprine [8, 9], methotrexate [10], anti-IL1 (anakinra) [11], and 2-chlorodeoxyadenosine [12]. We report a 12-year-old girl diagnosed with XD and treated with seven courses of 2-chlorodeoxyadenosine (2CdA) leading to relief of pain and resolution of mucosal, central nervous system, and cutaneous lesions, with subsequent restoration of menstrual cycles.

## 2. Case Presentation

A 12-year-old girl, withoutsignificant past medical history, presented with numerous asymptomatic, firm, red-brown to yellowish papules, which were symmetric and predominantly on her face and axillary, inframammary, and inguinal folds. In addition, she recently developed amenorrhea, having started menses at ten years of age. Family history was significant for maternal hypertension, paternal grandfather with brain cancer, and maternal and paternal grandmothers with diabetes mellitus, but no xanthogranulomas, histiocytic disorders, or thyroid issues in the family. A year later the rash became more widespread: the number of papules increased, coalescing into plaques on her eyelids, antecubital and popliteal fossa, and axillary, inframammary, and inguinal folds. A skin biopsy revealed a nodular infiltrate of macrophages, including multinucleated macrophages with peripheral foamy cytoplasm or Touton giant cells, consistent with xanthogranuloma disease. In the clinical context of the distribution of the cutaneous disease, the diagnosis of XD was made. Labs at this time showed a normal complete blood count, normal erythrocyte sedimentation rate, normal cholesterol, and normal lipid panel. She demonstrated slightly elevated triglycerides (172 mg/dL).

**Figure 1:**
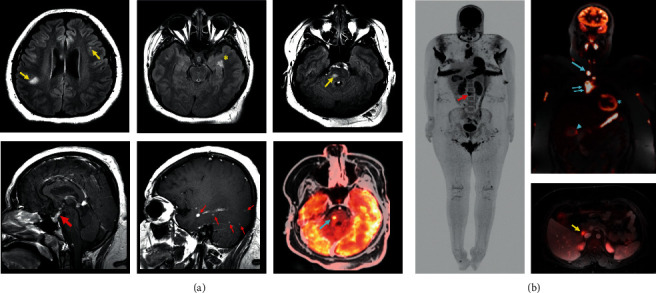
Disease status prior to intervention with 2CdA. (a) Brain MRI: multiple focal lesions are seen at the subcortical white matter and brainstem (yellow arrows), cerebellum (not shown), and left mesial temporal lobe/amygdala (^*∗*^) on T2 FLAIR imaging. Enhancing soft tissue is also seen at the pituitary stalk extending into the sella (big red arrow). Additional enhancing nodules (small red arrows) are seen in the brain. Corresponding abnormal FDG uptake (teal arrow) is also demonstrated from a brain image in the whole-body PET-MRI. (b) Whole-body PET MRI: multiple foci of abnormal FDG activity are seen in the neck, including the right thyroid (long teal arrow) and in the mediastinum along the thymic tissues (double teal arrows). Diffuse mucosal thickening of the gastric wall (^*∗*^) and gallbladder wall (arrowhead) with FDG-avidity also suggested possible XD involvement and/or inflammation. Enlarged and FDG avid portocaval lymph node (yellow arrow) and involvement of the vertebral body (red arrow) were also present.

Three months later she developed frequent urination, increased thirst, right-sided hearing loss, and gagging. Brain MRI and whole-body positive electron tomography (PET) demonstrated several CNS lesions and a thyroid lesion, and possible gastrointestinal system involvement, with increased metabolic activity (Figure 1). Pathology of the thyroid nodule showed bland histiocytic proliferation. Diabetes insipidus was diagnosed through history and urine studies, and she started anti-diuretic hormone with good response. Increasing cutaneous involvement of intertriginous areas and painful maceration in the inguinal folds led to difficulty in walking. Axillary maceration constrained her upper extremity range of motion which impeded activities of daily living. Ophthalmologic examination did not reveal ocular involvement. Significant laboratory results at this time included elevated C-reactive protein (13 mg/dL) and erythrocyte sedimentation rate (40 mm/hr), with a normal CBC, and further increase in triglycerides (227 mg/dL), but her cholesterol, HDL, LDL, and VLDL remained normal.

**Figure 2:**
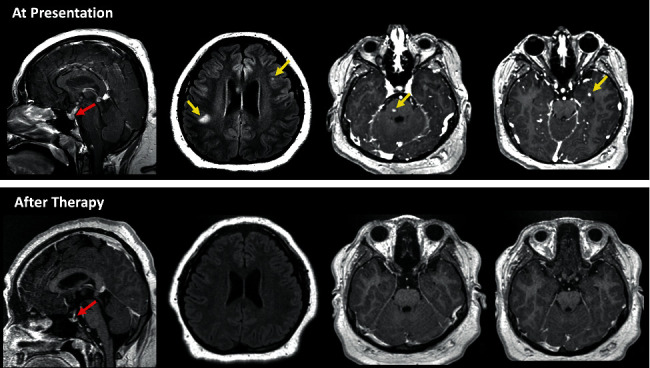
XD response to 2CdA on neuroimaging. MRI brain at presentation and comparison imaging after six cycles of 2CdA. Abnormal soft tissue at the pituitary stalk and extending into pituitary tissue has resolved after therapy (red arrows). Note that additional lesions in the supratentorial brain (yellow arrows) have also resolved after therapy.

**Figure 3:**
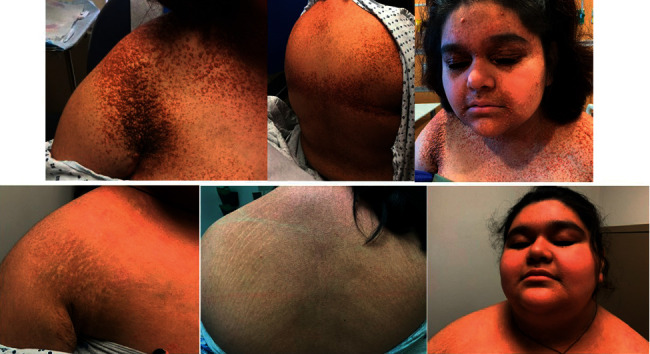
XD response to 2CdA on physical exam. Physical exam of characteristic involved regions just prior to starting 2CdA (top row). Physical exam of same regions at follow-up visit twenty months off-therapy (bottom row).

She began treatment with 2CdA at 5 mg/m^2^/day x five days given intravenously every twenty-eight days. Weekly sulfamethoxazole and trimethoprim for pneumocystis jiroveci pneumonia prophylaxis were prescribed (three consecutive days each week). Pain improved after cycle 1, and she had significant skin improvement after cycle 2. After six cycles, she had no pain of previously involved regions, and her cutaneous lesions greatly improved, with largely only residual post-inflammatory discoloration of face, arms, and intertriginous areas, in addition to resolution of intracranial lesions (Figure 2). At this time, however, her DI and amenorrhea persisted. She completed all cycles well without complications (i.e., febrile neutropenia, transfusions for anemia or thrombocytopenia, or nausea or vomiting). Because she demonstrated such an encouraging improvement after six cycles, two additional cycles of 2CdA to improve cosmesis were planned. Cycle 7 was dose reduced by 20% to 4 mg/m^2^/day x five days due to mild thrombocytopenia (99 K/uL). A repeat PET scan after cycle 7 demonstrated near complete resolution of all her metabolically active lesions except a small soft tissue lesion in the anterior mediastinum and another cutaneous lesion overlying the right breast. At the planned start of cycle 8, her platelet count was 61 K/uL, thus prompting discontinuation of additional 2CdA due to longitudinally dropping platelet counts. At ten months off-therapy, her platelet counts normalized and her menstrual cycles returned. As of twenty months off-therapy, her skin lesions continue to heal (Figure 3).

## 3. Discussion

The decision to use 2CdA as treatment for this patient with XD was based on a comprehensive literature review, tolerability, and provider experiences in LCH and nLCH [13, 14]. 2CdA is an adenosine deaminase-resistant purine nucleoside phosphorylated by deoxycytidine kinase [15]. Resting and proliferating lymphocytes and monocytes have a high ratio of deoxycytidine kinase to 5-nucleotidase, making 2CdA preferentially in the phosphorylated state [16,17]. The phosphorylated state of 2CdA causes deoxyribonucleic acid breaks, triggering nicotinamide adenine dinucleotide depletion, and then eventual cell death [18]. The known toxicity to monocytes in vitro provided the rationale for initial therapy in a patient with histiocytosis [19]. Histiocytic disorders were later confirmed to be myeloid neoplasms, providing further rationale for the utilization of 2CdA [20]. Recently, 2CdA has demonstrated efficacy for XD patients in several case series (Table 1).

Kherzi et al. published the first case series of XD patients treated with 2CdA, with all treated patients having a disease response after five to eight courses of 2CdA. In the case series, two had pituitary findings, but only one patient received 2CdA, and this patient had resolution of the pituitary lesion [12]. A 23-year-old patient was treated with eight cycles of 2CdA for cutaneous XD and central diabetes insipidus, with significant improvement of his cutaneous XD after 2CdA therapy [21]. A 19-year-old patient was treated for cutaneous XD with seven courses of 2CdA, and no new lesions had developed at two years of follow-up [22]. A 55-year-old patient with cutaneous and laryngopharyngeal XD demonstrated resolution of his laryngopharyngeal lesions after only three cycles of 2CdA [23]. In a 9-year-old patient with diffuse skin involvement, optic atrophy, diabetes insipidus, and several intracranial lesions, 2CdA treatment led to flattening of skin lesions, decrease in intracranial lesion size, and improvement in DI and vision [24]. In another case series of three patients, cutaneous and mucosal lesions resolved after five to nine cycles of 2CdA [25]. Lastly, in a 24-year-old patient with diffuse cutaneous involvement, DI, a nasopharynx mass, and a mass obstructing the choana requiring tracheostomy, six cycles of 2CdA over six months led to respiratory relief, and the tracheostomy was able to be removed [26].

In our patient, 2CdA demonstrated clinical response after the first cycle, and her CNS lesions resolved by the sixth cycle with documented radiology images. This case report and the existing medical literature support the use of 2CdA as first line treatment for XD and in particular for central nervous system disease.

## Figures and Tables

**Table 1:** Summary of published responses to 2CdA in xanthoma disseminatum.

Reference	Patient	2CdA regimen	Outcome	Toxicities
Khezri 2011 [12]	Series of 8 patients: All with diffuse skin involvement; 1 patient with additional pituitary and airway involvement; 1 patient with pituitary, adenoid, and soft palate involvement	5 out of 8 patients received treatment: 0.14 mg/kg/day x 5 days every 4 weeks x 5–8 cycles	All patients had substantial improvement in established lesions; no new lesions in follow-up period (3 months to 8 years)	Joint pain and night sweats in 1 patient; no serious adverse effects reported

Gupta 2016 [21]	23yo M with diffuse skin involvement and DI	0.14 mg/kg/day x 5 days every 1 month x 8 cycles	Stopped developing lesions after cycle 2; marked improvement after cycle 5	None reported

Adisen 2017 [22]	19yo F with diffuse skin involvement	0.14 mg/kg/day x 5 days every 1 month x 7 cycles	Resolution of many lesions; residual hyperpigmented macules remaining; no new lesions in 2-year follow-up	None reported

Briones 2018 [23]	55yo M with diffuse skin and laryngopharyngeal involvement	Presumed 0.14 mg/kg/day for 5 days, every 1 month X 3 cycles (6-8 cycles planned, stopped due to thrombocytopenia)	Clinical resolution of changes in voice, significant improvement in skin, lip, and gingiva; complete resolution of all lesions and no new lesions at 10 months follow-u	Thrombocytopenia (early termination of planned therapy)

Patra 2019 [24]	9yo M with diffuse skin, DI, optic atrophy, and multiple intracranial lesions	0.14 mg/kg/day *x* 5 days every 1 month *x* 6 cycles	30–40% flattening of skin lesions, decrease in intracranial lesion size, and improvement in diabetes insipidus and vision	Asymptomatic transient leukopenia

Tuan 2019 [25]	3 patients all with diffuse skin involvement and DI; 1 patient with respiratory involvement requirement tracheostomy; 1 patient with bulbar involvement	0.14 mg/kg/day x 5 days every 1 month x 5–9 cycles	Significant resolution of cutaneous and mucosal lesions; no signs of relapse after 52–66 months of follow-up	None reported

Al-Tarcheh 2020 [26]	24yo F with diffuse skin, DI, tracheal, knee joint, and nasopharynx involvement	0.14 mg/kg/day x 5 days every 1 month x 6 cycles	Resolution of nasopharynx mass and tracheal lesion; no new skin lesions but no regression of established lesions	None reported

## Data Availability

Data is restricted for patient privacy. Consents for deidentified photographs and radiologic images have been obtained.

## Abbreviations

XD: Xanthoma disseminatum
nLCH: Non-Langerhans cell histiocytosis
DI: Diabetes insipidus
CNS: Central nervous system
LCH: Langerhans cell histiocytosis
2CdA: 2-Chlorodeoxyadenosine
PET: Positive electron tomography.
